# Genome-wide copy number variant screening of Saudi schizophrenia patients reveals larger deletions in cases versus controls

**DOI:** 10.3389/fnmol.2023.1069375

**Published:** 2023-02-10

**Authors:** Mahdi S. Abumadini, Kholoud S. Al Ghamdi, Abdullah H. Alqahtani, Dana K. Almedallah, Lauren Callans, Jumanah A. Jarad, Cyril Cyrus, Bobby P. C. Koeleman, Brendan J. Keating, Nathan Pankratz, Amein K. Al-Ali

**Affiliations:** ^1^Department of Psychiatry, King Fahd Hospital of the University, Al-Khobar and College of Medicine, Imam Abdulrahman Bin Faisal University, Dammam, Saudi Arabia; ^2^Department of Physiology, College of Medicine, Imam Abdulrahman Bin Faisal University, Dammam, Saudi Arabia; ^3^Department of Surgery, University of Pennsylvania School of Medicine, Philadelphia, PA, United States; ^4^Department of Clinical Biochemistry, College of Medicine, Imam Abdulrahman Bin Faisal University, Dammam, Saudi Arabia; ^5^Department of Genetics, Division Lab, University Medical Center Utrecht, Utrecht, Netherlands; ^6^Department of Laboratory Medicine and Pathology, University of Minnesota, Minneapolis, MN, United States

**Keywords:** schizophrenia, CNV, genomewide, variant, Saudi

## Abstract

**Introduction:**

Genome-wide association studies have discovered common polymorphisms in regions associated with schizophrenia. No genome-wide analyses have been performed in Saudi schizophrenia subjects.

**Methods:**

Genome-wide genotyping data from 136 Saudi schizophrenia cases and 97 Saudi controls in addition to 4,625 American were examined for copy number variants (CNVs). A hidden Markov model approach was used to call CNVs.

**Results:**

CNVs in schizophrenia cases were twice as large on average than CNVs in controls (*p* = 0.04). The analyses focused on extremely large >250 kilobases CNVs or homozygous deletions of any size. One extremely large deletion was noted in a single case (16.5 megabases on chromosome 10). Two cases had an 814 kb duplication of chromosome 7 spanning a cluster of genes, including circadian-related loci, and two other cases had 277 kb deletions of chromosome 9 encompassing an olfactory receptors gene family. CNVs were also seen in loci previously associated with schizophrenia, namely a 16p11 proximal duplication and two 22q11.2 deletions.

**Discussion:**

Runs of homozygosity (ROHs) were analyzed across the genome to investigate correlation with schizophrenia risk. While rates and sizes of these ROHs were similar in cases and controls, we identified 10 regions where multiple cases had ROHs and controls did not.

## 1. Introduction

Schizophrenia is a chronic mental disorder characterized by severe cognitive, behavioral, and emotional symptoms, with an estimated lifetime risk of approximately 1% in the general population. Despite pharmacological treatments for schizophrenia, associated disease progression remains high (Lieberman et al., 2005). Since the advent of antipsychotic medications, which target the type 2 dopaminergic receptor (DRD2) system, no new significant improvements in outcomes have been observed (Morgan et al., 2014). Elucidating the pathophysiology of schizophrenia is a necessary step in improving disease outcomes.

Schizophrenia is highly heritable. Several large genome-wide association (GWAS) and exome sequencing studies have unambiguously demonstrated that both common and rare genetic risk variation explain a considerable proportion of this heritability, and are linked to a range of disease-related factors, including susceptibility, progression, severity, and treatment response (Schizophrenia Working Group of the Psychiatric Genomics Consortium, 2014; Howrigan et al., 2020; Kushima et al., 2022). In particular, rare copy number variations (CNVs) have been implicated as significant risk factors. While individually these CNVs are rare, they confer a significant increase risk for disease (ORs of more than 2 and as high as 60; Giaroli et al., 2014; Nakatochi et al., 2021). When combined, such rare structural variants show enrichment of heritable and *de novo* CNVs in cases versus controls. Because most CNVs are rare in the population and show a high degree of heterogeneity, it remains difficult for even the largest studies to have significant power to detect genomewide significance for single CNVs. The Psychiatric Genomics Consortium (PGC) performed the largest GWAS and CNV studies to date including more than 20,000 people with schizophrenia. The GWAS for identification of common inherited variation identified 108 genetic loci associations impacting schizophrenia etiology (Schizophrenia Working Group of the Psychiatric Genomics Consortium, 2014). Replication of these 108 loci and identification of novel loci in smaller studies, however, suggests that associations at other genes may provide insight into new mechanistic hypotheses (Ma et al., 2013, 2021; Pardiñas et al., 2018; Lam et al., 2019). A CNV study comprising 41,321 individuals (21,094 cases) had sufficient power to detect significant enrichment of CNV burden and genome-wide significant association for eight loci: 1q21.1, 2p16.3 (*NRXN1*), 3q29, 7q11.2, 15q13.3, distal 16p11.2, proximal 16p11.2 and 22q11.2. The majority of GWAS and CNV studies have been conducted on European populations (Schizophrenia Working Group of the Psychiatric Genomics Consortium, 2014). Studying European populations alone greatly limits the ability to discover the causal variants of varying frequencies that underpin mechanisms of debilitating diseases, including schizophrenia. A cross-disorder CNV study combined and compared the CNV analysis of schizophrenia, bipolar disorder, and autism spectrum disorder in 8708 Japanese sample. This study confirmed the enrichment of CNVs in schizophrenia and found interesting differences between the disorders. Similarly, Lam et al., conducted a GWAS in East Asian populations and identified 176 genetic loci, replicating 89 of the 108 previously reported SNPs (Lam et al., 2019). Compared to European populations, high genetic correlation was observed, suggesting that the genetic basis of disease is shared between populations.

Few studies have been performed that include individuals from the Kingdom of Saudi Arabia. Therefore, we aimed to investigate whether we could confirm the relevance of known common and rare genetic risk variants in 136 Saudi Arabian schizophrenia patients as compared to controls (97 Saudi and 4,625 American). More importantly, we aimed to investigate whether the rare risk CNVs detected in the European and Asian populations are also present in our cohort.

## 2. Materials and methods

### 2.1. Patient selection

This case–control study was conducted on 136 Saudi patients with clinically diagnosed schizophrenia recruited from the Department of Psychiatry, King Fahd Hospital of the University (KFHU), Al-Khobar and Al-Amal Mental Health Complex, Dammam, both in the Eastern Province of Saudi Arabia. Schizophrenia was diagnosed according to the Classification of Mental and Behavioral Disorders Clinical Descriptions and Diagnostic Guidelines (10th revision) World Health Organization (WHO). This study was approved by the Ethical Committee of Imam Abdulrahman bin Faisal University and was conducted in accordance with the 1964 Helsinki Declaration and its later amendments. Informed written consent in English, with a verified translation in Arabic, was obtained from all participants in accordance with the Institutional Review Board (IRB # 2017-01-051). The control cohort consisted of 97 Saudi Arabian individuals and 4,625 American individuals. The United States controls consist of participants from iGeneTRAiN, which contain DNA from kidney allograft donors and kidney recipients and were genotyped using the same genotyping array in the same lab and processed together. The genome-wide genotyping array, quality control pipelines and cohorts have been described extensively before (International Genetics and Translational Research in Transplantation Network (iGeneTRAiN), 2015; Li et al., 2015).

### 2.2. DNA and genome-wide genotyping

Blood samples (5 ml) for DNA extraction were collected in EDTA tubes from Saudi schizophrenia patients and Saudi controls after obtaining signed informed consent. Genomic DNA was extracted from peripheral blood mononuclear cells using standard procedures and stored at −80°C (Qiagen, United States). DNA concentrations and purity were estimated by fluorometry using a NanoDrop 2000 Spectrophotometer (Thermo Fisher Inc., Massachusetts, United States) and were diluted to 20 ng/μl for genome-wide genotyping. Genome-wide genotyping was performed using the Axiom SNP tx v1 array (Thermo Fisher, USA) which comprises over 780,000 genetic variant and CNV probes (Li et al., 2015). Raw intensity files (Affymetrix CEL files) were processed with the Affymetrix Power Tools (version 1.19.0) following the best practice workflow.

### 2.3. Quality control of samples

The raw intensity data used to call genotypes is repurposed to identify CNVs. If a drop in intensity is identified for markers that are physically close together in the genome, then this is indicative of a deletion in the DNA. The reciprocal increase in intensity indicates that a duplication is likely present. The intensity metric (log R ratio or LRR) is computed by taking the sum of the probe intensity for each of the two alleles (X and Y) and normalizing it to the population using standard formulas (Peiffer et al., 2006). The percentage of alleles that are the B allele (B allele frequency or BAF) is another useful metric for calling CNVs. However, if either value deviates too much due to low quality DNA or sample failure, then it can lead to spurious CNV calls. Therefore, any samples with values that were more than three standard deviations from the mean for LRR (only for values between −2 and +2, so as to exclude regions with real copy number variation) or for BAF (only for markers with putative heterozygous genotypes, i.e., values between 0.15 and 0.85) were excluded. All samples were required to have a call rate greater than 95%. Analysis was performed on samples that were verified to be unrelated based on PI_HAT<0.125 with all other individuals, as calculated by the --genome option implemented in PLINK (Purcell et al., 2007).

Intensity data for all high-quality samples were then used to validate sex and to search for individuals with sex aneuploidy. These can be identified by a split BAF pattern that extends for megabases at a time. Genvisis identifies these using a hidden Markov model similar to the CNV calling algorithm but that focuses exclusively on the BAF distribution. Chromosomal arms with large events can also be identified by plotting the standard deviation of the BAF for heterozygous markers (values 0.15 to 0.85) on one axis and the interquartile range (IQR) on the other. Finally, the SNPs that were used to call runs of homozygosity passed all marker QC, including genotype call rate > 95%, Hardy Weinberg *p*-value > 0.00001, PLINK mishap *p*-value > 0.00001, and differential missingness or frequency by sex (*p* > 0.0001). In addition, markers were pruned to remove markers that were in strong linkage disequilibrium (LD; PLINK --indep-pairwise 50 5 0.2) to exclude common segments that appear as ROHs but that are more likely to have arisen from LD (Gibson et al., 2006; Howrigan et al., 2011). These same variants were used to perform a principal components analysis that captures ancestry using the Genvisis software version 0.1.16 (https://www.genvisis.org/).

### 2.4. CNV calling and filtering

Autosomal CNVs were called using Genvisis (genvisis.org; Skidmore et al., 2016), which is modeled after the PennCNV algorithm (Wang et al., 2007) with the following caveats. First, the LRR values for a set of probes that map uniquely to the genome and that do not commonly deviate from a copy number of two were used in a principal component analysis (PCA) to identify DNA quantity and quality effects (PCs 1 and 2, respectively) and to identify any batch effects that may have caused a shift in the intensity clusters (PCs 3–10). These effects were then regressed out of the LRR values to create more stable estimates of copy number for each probe. Data were then GC-wave adjusted and fed into the hidden Markov model (HMM) in Genvisis to call CNVs. CNV calls were then filtered for higher quality, with the minimum number of probes required for a CNV call being three for homozygous deletions, and 15 for heterozygous deletions or duplications. The confidence threshold for CNV calling was set to a Bayes factor of 10. Adjacent CNV calls were merged if they had the same copy number and if the distance between them was <20% of the smaller CNV call. If a CNV call spanned the centromere of a chromosome, it was split into two calls, with the first one ending at the last probe before the centromere and the second one beginning at the first probe after the centromere.

### 2.5. CNV selection and prioritization

We then used PLINK to perform segmental analysis to identify variants that were present in the Saudi schizophrenia cases but not in the Saudi controls using the --mperm 10,000 option. This analysis randomly permutes the case/control status of the individuals 10,000 times and determines how many times CNVs overlap a particular locus in each replicate. This generates an empirical value of p that relays how often the observed number of cases overlapping a particular locus might be by chance. We followed up on these variants to confirm that they were rare among the in-house controls. Permutation of the phenotype (1 million replicates) was used to determine statistical significance.

### 2.6. Runs of homozygosity

PLINK was also used to identify regions where 100+ consecutive markers have homozygous genotypes, more than by chance alone. Variants with an allele frequency less than 1% were excluded from the ROH analyses. Since linkage disequilibrium (LD) can similarly confound these analyses, markers were pruned such that no two markers had a pairwise r^2^ value greater than 0.30. Regions were then identified (using the PLINK parameter in parentheses) that were (1) at least one megabase (Mb) in size (--homozyg-kb 1,000), (2) spanned at least 100 SNPs (--homozyg-snp 100), (3) had a density of at least one SNP per 50 kb (--homozyg-density 50), (4) had no more than one variant per megabase having a heterozygous genotype (--homozyg-window-het 1), and (5) had no gap larger than 1 Mb (e.g., overlapping at centromere; --homozyg-gap 1,000).

## 3. Results

**Figure 1:**
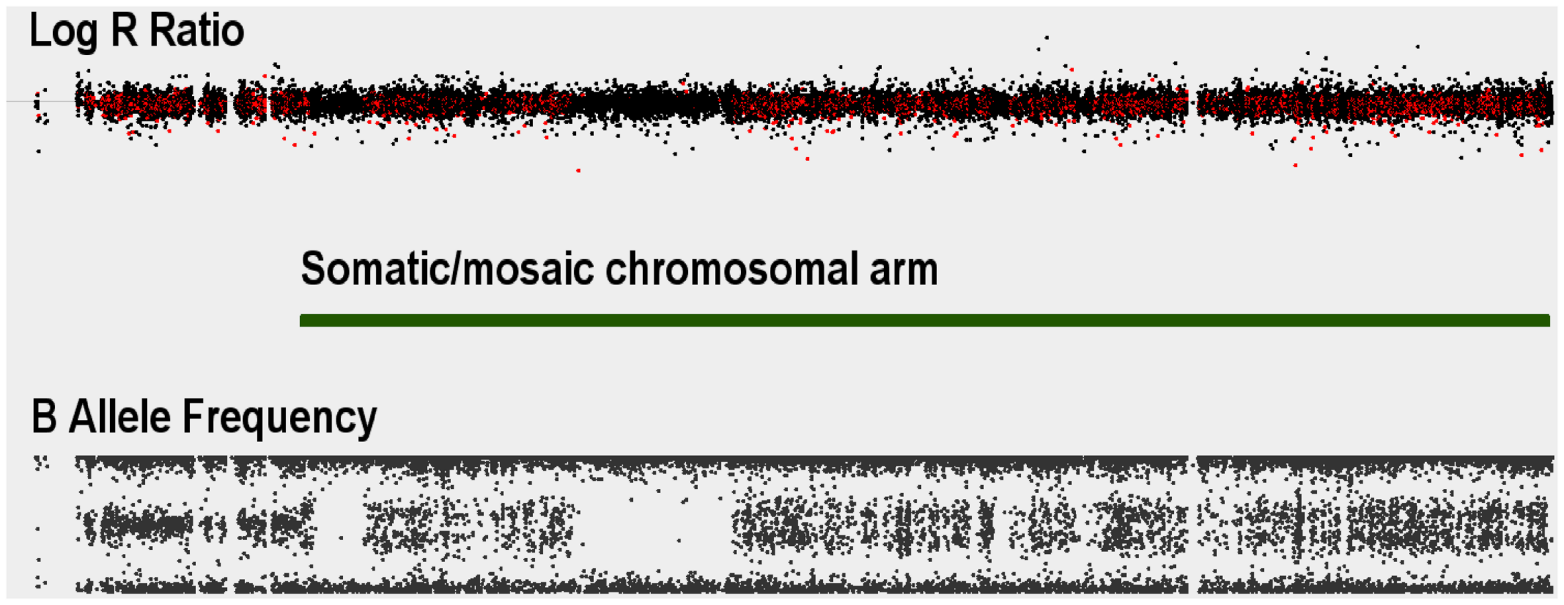
Autosomal somatic copy number variant example for a Saudi schizophrenia case: Log R Ratio (LRR) and B-allele frequency (BAF) values for a case with a somatic event spanning almost the entire length of chromosome 15q. The split in the BAF distribution starting at where Genvisis called the breakpoints for the mosaic event (green bar).

**Figure 2:**
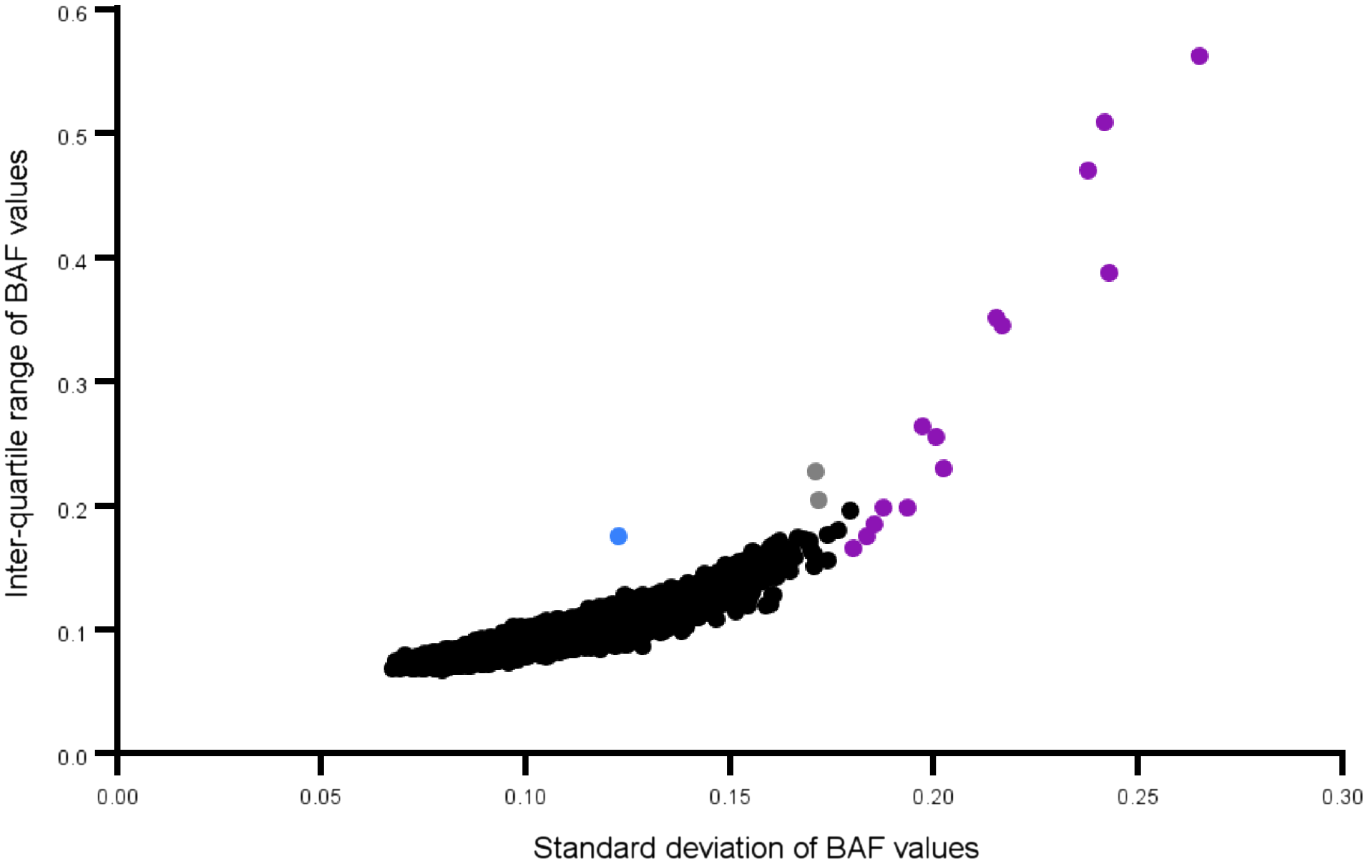
Mosaicism plot of all Saudi schizophrenia cases and controls: Each dot represents a chromosomal arm of an individual. Outliers for standard deviation and the interquartile range of the BAF values, are an effective way to screen for large mosaic events. The chr15q arm is shown in blue. The dots in purple are chromosomes where the entire arm or close to the entire arm is a long run of homozygosity. Only those B Allele Frequency (BAF) values between 0.15 and 0.85 are used in these calculations.

Demographic and clinical characterization of patients and controls are presented in Table 1. High quality data were available for 136 Saudi cases (130 male) and 97 Saudi controls (57 male), and for 4,625 common controls genotyped on the same genome-wide genotyping array platform and subjected to the same genotype and CNV analyses. Principal components analysis of the genotypes confirmed the ancestry of the Saudi cases and controls to be from Southwest Asia (see Supplementary Figure S1). One male sample had a marked partial loss of chromosome Y, which is the most common genetic aberration in men (Danielsson et al., 2020). This control male (SZ-C^−100^) was 50 years of age at DNA collection and the CNV loss is illustrated in Supplementary Figure S2 in which the median intensities for the sex chromosomes are plotted. No other samples had aneuploidy of a sex chromosome (no Klinefelter, Turner or Triple X syndromes, mosaic or complete, were observed). One individual (SZ-P-129) had an autosomal mosaic chromosomal arm that extended for 85% of chr15q (Figure 1), evidenced by the modest split in the intermediate BAF band and no deviation of the corresponding LRR. Intriguingly, two stretches of homozygosity were proximal to this pattern. The plot of BAF standard deviation (SD) and interquartile range (IQR) did not identify any other samples with a mosaic event, although individuals with large runs of homozygosity were identified (Figure 2).

**Table 1:** Demographic and clinical characterization of patients and controls.

Parameter	Patients	Controls
Number of patients	136	97 (+4,722 US)
Male (%)	78.3	44.1
Age ± SD (year)	38 ± 10.6	50.9 ± 7.9
Type of Schizophrenia (N)		
Paranoid schizophrenia (%)	105 (77.2)	
Residual schizophrenia (%)	2 (1.5)	
Schizophrenia, unspecified (%)	22 (16.2)	
Schizoaffective manic type (%)	6 (4.5)	

Analysis of all 233 Saudi case and control individuals resulted in a total of 1,590 autosomal CNV calls that passed inclusion filtering criteria. The patient cohort had similar numbers of CNVs as the control group, but the average length of each CNV in the patient cohort was larger (125.7 kb compared to 86.15 kb), and therefore a larger proportion of the genome in the patient cohort was affected. However, these differences were not significantly different in our modestly sized cohort (empirically derived *p*-value = 0.32). This same pattern became significant when restricting the variants to those that were larger than 100 kb, where cases averaged 415.4 kb per variant and controls averaged 222.1 kb per variant (*p* = 0.036) as shown on Table 2.

**Table 2:** Number of samples and events and a comparison of the rate of events.

Parameter	Copy number variants (CNVs) that are >100 kb			Runs of Homozygosity (ROHs)			Cases	Controls	*p*-value	Cases	Controls	*p*-value
Number of individuals	136	97		136	97	
Number of events	197	164		1884	1,357	
Rate of events	1.459	1.673	0.918	13.96	13.85	0.475
Proportion of individuals with at least on event	75.6%	81.6%	0.899	94.8%	93.9%	0.486
Total amount of genome affected	642.8 kb	497.6 kb	0.326	9.2 Mb	9.1 Mb	0.480
Average size of event	415.4 kb	222.1 kb	0.036	5.1 Mb	5.0 Mb	0.370

**Figure 3:**
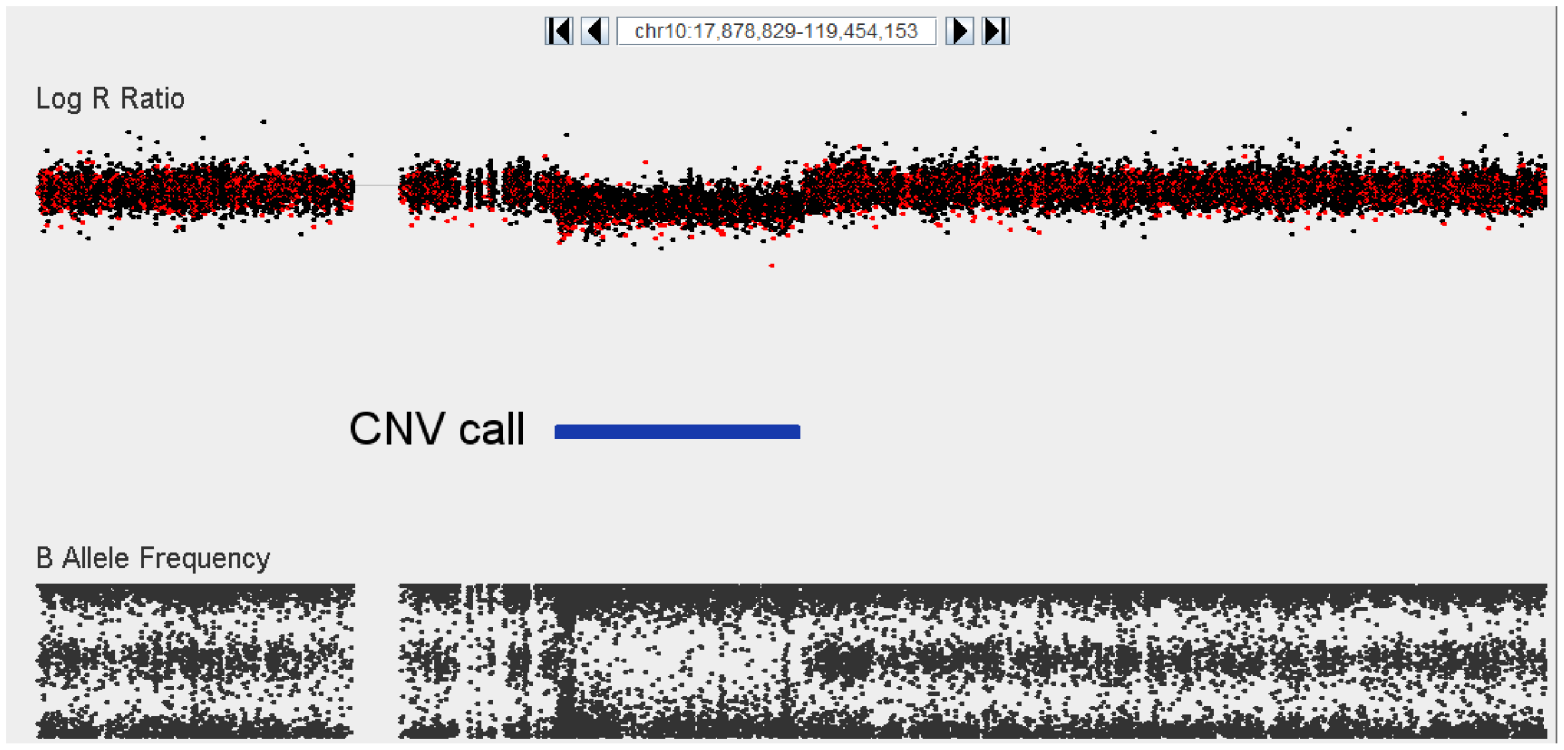
Visualization of a 16.5 Mb deletion at chromosome 10 (52,931,142–69,418,051) encompassing at least 68 different genes. The top panel depicts logR ratio data (signal intensity) and the bottom panel depicts the B-allele frequency (ratio of minor to major alleles). Dots indicate the location of SNPs on the array. CNV calls made using the Genvisis algorithm are indicated using the blue colored bar between the plots.

The segmental analysis did not reveal any variants at genome-wide significance (EMP2 < 0.05); however, two candidate regions were identified where a similarly sized CNV was present in more than one case, absent in the Saudi controls, and at least extremely rare in the in-house controls. These two regions on chromosome 7 and 9, respectively, and the affected genes are shown in Table 3. In addition, there was a particularly notable singleton CNV that was observed in only one individual. This sample harbors a 16.5 megabase deletion on chromosome 10 (Figure 3). This deletion (chr10:52,931,142-69,418,051) spans at least 68 genes (listed in Supplemental Table S1), and consequently, it is likely pathogenic according to the ACMG recommendation. The next largest deletion observed is 1.4 Mb, which is similarly predicted to be pathogenic. Finally, 17 CNVs had previously been associated to schizophrenia in a large GWAS. In our relatively small cohort, we detected only one of these 17 CNVs, namely the 16p11, proximal duplication in a single case, and to cases with 22q11.2 deletions.

**Table 3:** Copy Number Variants present in more than one Saudi Schizophrenia case, zero matched controls, and rare in the American controls.

Region	Type/Size	Number of cases (%)	Number of controls (%)	Number of in-house controls (%)	Genes affected
chr7:16,831,346-17,645,439	Duplication 814 kb	2 (0.8%)	0 (0.0%)	2 (0.04%)	AGR2, AGR3, AHR, LOC102659288, LOC101927630
chr9:107,220,263-107,497,215	Deletion 277 kb	2 (0.8%)	0 (0.0%)	0 (0.0%)	OR13F1, OR13C4, OR13C3, OR13C8, OR13C5, OR13C2, OR13C9, OR13D1

Genes in these CNV regions are also listed.

A large number of ROHs were identified in both cases and controls, and a skew was not found in either direction for number, rate, proportion, total affected or average size (Table 4). We identified five regions where 10 or more cases had an ROH overlapping a particular region. We then extended that region in both directions until fewer than seven cases were overlapping the flanking regions, or a new control was seen to have an ROH. In addition to these five regions, we also identified five regions that had at least seven cases and zero controls with an ROH in that region. Each region and the number of individuals with an ROH in that region are delineated in Table 5.

**Table 4:** Number of cases and controls with samples and events >100 kilobases in known regions.

Locus	UCSC_hg19	CNV type	Direction	#Case	#Cntrl	Reference
1q21.1	chr1:145,934,643-147,709,376	Loss and gain	Risk	0	8	Levinson et al. (2011)
2p16.3 NRXN1	chr2:50,147,488-51,259,674	Loss	Risk	0	0	Viñas-Jornet et al. (2014)
3q29	chr3:195,745,603-197,355,603	Loss	Risk	0	0	Levinson et al. (2011) and Mulle et al. (2010)
7q11.21 ZNF92	chr7:64,838,768-64,865,998	Loss and gain	Protective	0	54	Marshall et al. (2017)
7q11.23	chr7:72,742,064-74,142,064	Gain	Risk	0	0	Mulle et al. (2014)
7p36.3 VIPR2 WDR60	chr7:15,8453,198-15,897,237	Loss and gain	Risk	0	0	Levinson et al. (2011) and Vacic et al. (2011)
8q22.2 VPS13B	chr8:100,025,494-100,889,808	Loss	Risk	0	0	Marshall et al. (2017)
9p24.3 DMRT1	chr9:841,690-969,090	Loss and gain	Risk	0	2	Marshall et al. (2017)
13q12.11 ZMYM5	chr13:20,411,593-20,437,773	Hain	Protective	0	3	Marshall et al. (2017)
AS/PWS	chr15:2,482,000-2,843,000	Gain	Risk	0	0	Rees et al. (2014a)
15q11.2	chr15:22,798,636-23,088,559	Loss	Risk	0	19	Cox and Butler (2015)
15q13.3	chr15:31,132,708-32,482,708	Loss	Risk	0	1	Sahoo et al. (2011)
16p13.11	chr16:1,551,000-1,630,000	Gain	Risk	0	0	Ramalingam et al. (2011)
16p11.2 (distal)	chr16:28,822,499-29,052,499	Loss	Risk	0	2	Guha et al. (2013)
16p11.2 (proximal)	chr16:29,652,499-30,202,499	Gain	Risk	1	1	Dall’Olio et al. (2014)
17p12	chr17:1,416,000-1,543,000	Loss	Risk	0	3	Kirov et al. (2009)
17q12	chr17:3,481,000-3,620,000	Loss	Risk	0	3	Moreno-De-Luca et al. (2011)
22q11.21	chr22:19,020,000-21,420,000	Loss	Risk	2	0	Bassett and Chow (2008)
22q11.21	chr22:19,020,000-21,420,000	Gain	Protective	0	3	Rees et al. (2014b)

**Table 5:** A list of regions with runs of homozygosity (ROH) in multiple Saudi Schizophrenia cases where few to zero are observed in controls.

Region	minAFF	maxAFF	numUNAFF
chr11:41,633,193-43,738,673	8	9	0
chr11:92,507,397-97,753,229	7	10	1
chr11:100,189,226-103,910,105	8	13	2
chr13:43,541,285-48,716,219	7	10	0
chr14:25,142,208-48,716,219	7	8	0
chr14:49,590,913-54,191,445	8	10	0
chr14:61,894,646-62,670,326	7	7	0
chr14:99,840,955-103,644,765	8	14	1
chr15:100,328,822-101,076,889	8	8	0
chr20:51,011,788-51,712,879	7	7	0

The maximum number of individuals with schizophrenia and an ROH (maxAFF) at any point in that region, as well as the minimum number of affected individuals at any given point in that region (minAFF) are indicated, as is the number of unaffected individuals with ROH events (numUNAFF).

## 4. Discussion

Several large association studies and clinical genetic studies have unambiguously shown the importance of CNVs, including both deletions and duplications, in understanding the pathology of schizophrenia. Furthermore, detection of a relevant pathogenic CNV can aid in diagnosis and counselling of patients. Recognizing the lack of data for schizophrenia cases with a specific Arabic ethnicity, we set out to assess the contribution of CNVs to the genetic risk of disease in the Saudi population. In our cohort of 136 schizophrenia cases, we detected one likely relevant variant and evidence for an increased average size of CNV compared to controls. These observations suggest that CNVs have putative biological relevance in the population of Saudi Arabia.

In our cohort of cases, there were three large CNVs encompassing loci previously associated with schizophrenia: one duplication of 16p11 (proximal) and two deletions in 22q11.2. Both of these are in “hotspot” where structural variants frequently form *via* non-allelic homologous recombination (NAHR) that is mediated by the repetitive DNA on either side of the breakpoints that are highly homologous (Kirov et al., 2009; Goldenberg, 2018). The 16p11 deletion has been robustly associated with psychiatric disorders, including intellectual disability, attention deficit hyperactivity disorder (ADHD), and autism spectrum disorder (Niarchou et al., 2019). 22q11.2 deletion syndrome (also known as DiGeorge syndrome) can lead to a variety of phenotypes including heart defects, cleft palate, hearing loss, intellectual disability, and delayed growth or speech development.

**Figure 4:**
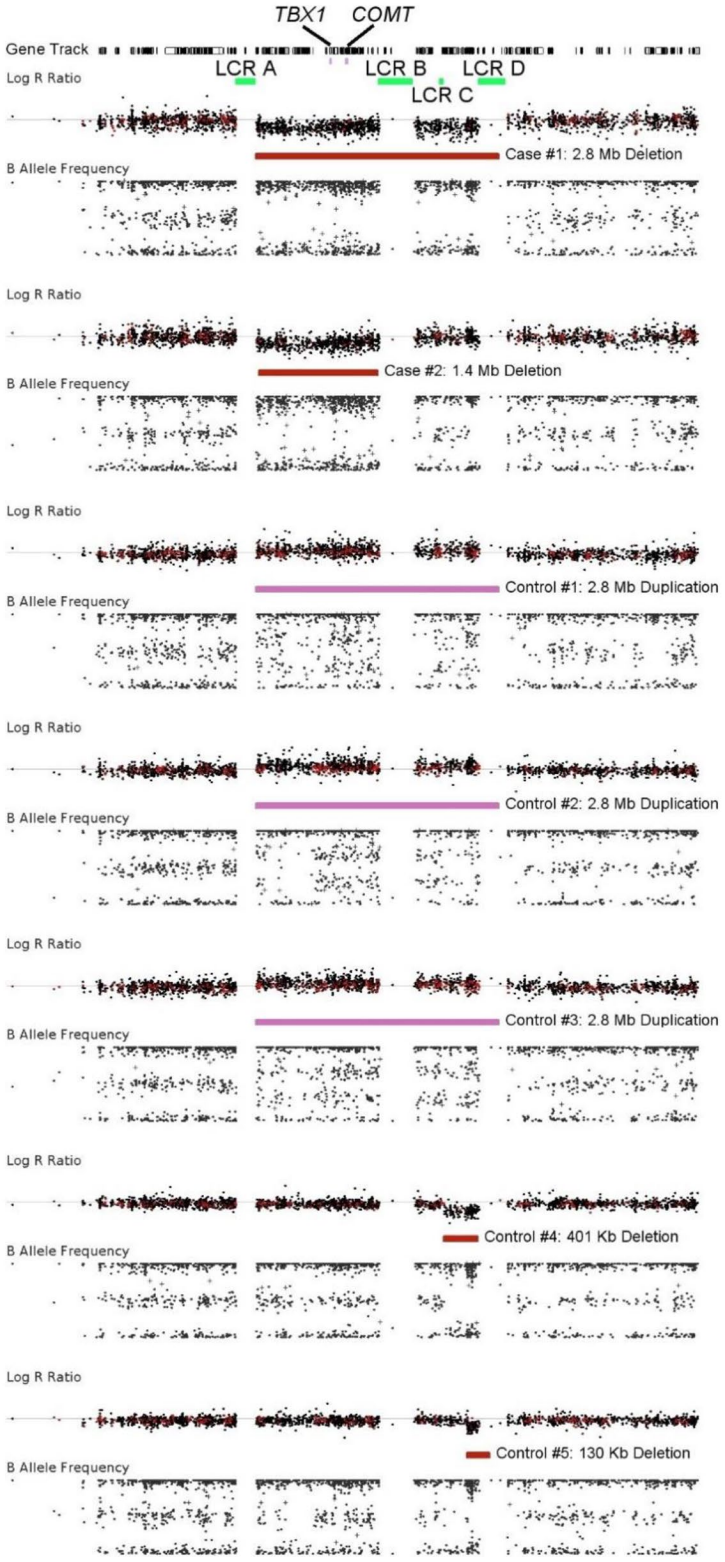
Visualization of a CNVs identified at the chromosome 22q11.2 region. The top panel for each individual depicts logR ratio data (signal intensity) and the bottom panel depicts the B-allele frequency (ratio of minor to major alleles). Dots indicate the location of probes on the array. The CNV calls were made using Genvisis and colored red for deletions and pink for duplications. These CNVs are thought to be created due to non-allelic homologous recombination at the low copy repeats (LCRs) in green. Most are between LCR A and LCR D, but the deletion in Case #2 is between LCR A and LCR B. The most critical genes causing the characteristics of 22q11.2 deletion syndrome are thought to be *TBX1* and *COMT*. Duplications of this region are protective against schizophrenia.

Which characteristics are present in a 22q11.2 deletion case may have to do with which genes are deleted. There are multiple low copy repeats (LCRs) in the region leading to multiple permutations of which genes are deleted. Most have a deletion of the full ~3 megabase region, but a “critical region” has been identified that includes the *TBX1* and *COMT* genes (Weksberg et al., 2007). Our own two cases corroborate this pattern, with both deletions spanning this region and Case #2 (Figure 4) having a far shorter deletion. Moreover, we see two smaller deletions in controls that are for only a small part of the distal region (Figure 4) that do not overlap this critical region. In addition to deletions, duplications of this same region are noted in the general population and have been associated with protection against schizophrenia. In our study, we see three such duplications of the entire ~3 Mb region, all in controls (Figure 4).

Besides those at the known loci, only one likely pathogenic CNV was detected, the 16.5 Mb deletion on chromosome 10 that was detected in one patient that involved deletion of 68 genes. One patient showed evidence of a mosaic CNV involving 85% of the long arm of chromosome 15 (15q). This mosaic CNV overlaps with the recurrent 15q13.3 deletion that is robustly associated with schizophrenia. The signal from this case showed evidence for low-grade mosaicism, while it was unclear from the LRR whether this CNV is a duplication or a deletion, and therefore its clinical relevance remains unclear. Indeed, large somatic/mosaic chromosomal aberrations (mCa), including somatic deletions and duplications that span almost an entire chromosome, are common in older individuals (~2% of individuals 85 years and older) but rare at younger ages.

These cases illustrate that known recurrent or other clinically relevant CNVs are also present in the Saudi population. One case presented a partial loss of the Y-chromosome that is observed frequently and associated with advanced age, which is likely unrelated to schizophrenia. Therefore, the percentage of clinically relevant CNVs in our sample is slightly lower (~3%) as compared to that of previous studies that have mainly been performed in populations of European ancestry, which range between 6 and 8% (Costain et al., 2013; Daghsni et al., 2018). This observation may suggest that population specific factors, either genetic or non-genetic, may contribute to the disease risk in the Saudi population.

As can be expected for our relatively modest sample size, we could not detect an overall significant increase in CNV burden in cases versus controls. This is not surprising as it is well-known that although CNVs are robustly associated with high effect sizes with schizophrenia, they are present at low frequencies in cases. Indeed, the total number of CNVs observed in our case cohort was similar to that in controls. However, the average size of CNVs carried by cases was significantly larger than that of controls. Such an observation has been reported before and may relate to the probability that larger CNVs involve more genes than smaller CNVs, thus increasing the likelihood of pathogenicity. One limitation of our study is that most of our controls are from a slightly different ethnic background. It’s possible that certain CNVs are more common in one population or another. That said, we had almost as many ethnically matched controls as we did cases, and the variants we did find were not present in those individuals, suggesting that they are not common in the Saudi population.

Finally, we observed a large number of regions showing a run of homozygosity in both cases and controls with no evidence of significant differences. Unlike a putatively causal CNV, a ROH is not thought to be causal in and of itself. It is merely an indication of the possibility that a rare recessive variant(s) may be found in the homozygous state in this region. For this reason, we favored regions where there were relatively few unaffected ROH events, but we did not require it when there were >10 cases.

Overall, our study shows evidence for an involvement of CNVs in the risk for schizophrenia in the population of Saudi Arabia. However, we cannot confirm that the rare, yet recurrent CNVs that are robustly associated in Western European populations, also contribute significantly to the risk of schizophrenia in this population. Further studies involving an increased sample size are needed to provide sufficient power to detect novel CNVs that may be unique to this population. Such a study would determine the benefit of genetic screening for clinically relevant CNVs to aid in diagnosis, prognosis, and management of schizophrenia. These data show a potential role of large CNVs as risk factors for schizophrenia in Saudi cases, and investigation into the genes that they affect may provide further insights into the etiology of schizophrenia in these populations.

## Data availability statement

The data presented in the study are deposited in the https://www.ebi.ac.uk/ena/browser/view/PRJEB59193 repository, accession number PRJEB59193.

## Ethics statement

The studies involving human participants were reviewed and approved by IRB Committee, Imam Abdulrahman Bin Faisal University. The patients/participants provided their written informed consent to participate in this study.

## Author contributions

MA, AA, DA, and JJ were involved in the design of the work, critically revising the protocol, patient recruitment, data acquisition, and interpretation of data and drafting of the manuscript. KG, CC, and AA-A were involved in the design of the work, critically revising of protocol, laboratory work, data acquisition, and interpretation of data and drafting of the manuscript. NP, LC, BKo, and BKe were involved in the design of the work, analysis, and interpretation of data and drafting of the manuscript. All authors contributed to the article and approved the submitted version.

## Funding

The research project was financially supported by the Deanship of Research at Imam Abdulrahman bin Faisal University.

## Conflict of interest

The authors declare that the research was conducted in the absence of any commercial or financial relationships that could be construed as a potential conflict of interest.

## Publisher’s note

All claims expressed in this article are solely those of the authors and do not necessarily represent those of their affiliated organizations, or those of the publisher, the editors and the reviewers. Any product that may be evaluated in this article, or claim that may be made by its manufacturer, is not guaranteed or endorsed by the publisher.
