# Supplementary material for: Genome-wide copy number variant screening of Saudi schizophrenia patients reveals larger deletions in cases versus controls
Source: Front Mol Neurosci. 2023 Feb 10;16:1069375. doi: 10.3389/fnmol.2023.1069375 (PMC9950097; doi:10.3389/fnmol.2023.1069375)
Supplement: Supplementary file 1 [file Table_1.docx]

**Supplementary Table 1:** The deletion chr10:52931142-69418051 spans at least 68 genes (listed below) and consequently, it is likely pathogenic according to the ACMG guidelines.

| **Gene Code** | **Gene Name** | **Gene type** | **Chromosomal Location** | | **Other Phenotype, disease and trait (Ensembl Database)** | **Schizophrenia Related Articles** | |
| --- | --- | --- | --- | --- | --- | --- | --- |
|  |  |  | **Cytogenetic** | **Chromosome: bp** |  | **PMID** | **doi:** |
| PRKG1 | protein kinase cGMP-dependent 1 | protein coding | 10q11.23-q21.1 | Chr 10: 50,990,888-52,298,423 | Aortic aneurysm, familial thoracic 8; Familial thoracic aortic aneurysm and aortic dissection | 23922650; 29257106 | 10.1371/journal.pone.0067776; 10.3390/ijms18122763 |
| MIR605 | microRNA 605 | ncRNA | 10q21.1 | Chr 10: 51,299,573-51,299,655 | - | - | - |
| LOC100419964 | Ras suppressor protein 1 pseudogene 3 | pseudogene | 10q21.1 | - | - | - | - |
| CSTF2T | cleavage stimulation factor subunit 2 tau variant | protein coding | 10q21.1 | Chr 10: 51,695,486-51,699,595 | - | - | - |
| LOC100506939 | PRKG1-AS1 PRKG1 antisense RNA 1 | ncRNA | 10q21.1 | Chr 10: 52,230,398-52,314,507 | Rheumatoid arthritis; Skeletal muscle aging | - | - |
| DKK1 | dickkopf WNT signaling pathway inhibitor 1 | protein coding | 10q21.1 | Chr 10: 52,314,281-52,318,042 | Arnold-Chiari malformation type I; Idiopathic juvenile osteoporosis | 29507296; 20153141 | 10.1038/s41398-018-0102-1; 10.1016/j.schres.2010.01.014 |
| RPL31P44 | ribosomal protein L31 pseudogene 44 | pseudogene | 10q21.1 | Chr 10: 52,389,112-52,389,488 | - | - | - |
| PRKRIRP3 | THAP domain containing 12 pseudogene 3 | pseudogene | 10q21.1 | Chr 10: 52,411,135-52,413,492 | - | -- | - |
| MBL2 | mannose binding lectin 2 | protein coding | 10q21.1 | Chr 10: 52,765,380-52,772,784 | Mannose-Binding Lectin Deficiency | 32279906; 34663707 | 10.1016/j.jocn.2020.04.001; org/10.17712/nsj.2021.4.20200050 |
| PCDH15 | protocadherin related 15 | protein coding | 10q21.1 | Chr 10: 53,802,771-55,627,942 | Autosomal recessive non-syndromic sensorineural deafness type DFNB; deafness, autosomal recessive 23; Usher syndrome type 1, 1D & 1F | 27058588 | org/10.1371/journal.pone.0153224 |
| LOC100420737 | NEFMP1 neurofilament medium pseudogene 1 | pseudogene | 10q21.1 | Chr 10: 54,599,678-54,600,683 | - | - | - |
| MIR548F1 | microRNA 548f-1 | ncRNA | 10q21.1 | Chr 10: 54,607,874-54,607,957 | - | - | - |
| MTRNR2L5 | MT-RNR2 like 5 | pseudogene | 10q21.1 | Chr 10: 55,599,042-55,600,728 | - | - | - |
| GAPDHP21 | glyceraldehyde 3 phosphate dehydrogenase pseudogene 21 | pseudogene | 10q21.1 | Chr 10: 55,667,341-55,668,350 | - | - | - |
| LOC100419872 | zinc finger MYM-type containing 4 pseudogene | pseudogene | 10q21.1 | - | - | - | - |
| ZWINT | ZW10 interacting kinetochore protein | Protein coding | 10q21.1 | Chr 10: 56,357,227-56,361,273 | - | 24564241 | 10.1186/1471-2164-14-S5-S10 |
| MIR3924 | microRNA 3924 | ncRNA | 10q21.1 | Chr 10: 57,304,479-57,304,559 | - | - | - |
| MRPS35P3 | mitochondrial ribosomal protein S35 pseudogene 3 | pseudogene | 10q21.1 | Chr 10: 57,982,285-57,982,993 | - | - | - |
| IPMK | inositol polyphosphate multikinase | protein coding | 10q21.1 | Chr 10: 58,191,517-58,267,894 | Hereditary neuroendocrine tumor of small intestine | 27088644 | 10.1001/jamaneurol.2016.0150 |
| CISD1 | CDGSH iron sulfur domain 1 | protein coding | 10q21.1 | Chr 10: 58,269,162-58,289,586 | - | - | - |
| UBE2D1 | ubiquitin conjugating enzyme E2 D1 | protein coding | 10q21.1 | Chr 10: 58,334,979-58,370,751 | - | 28226265; 25578144 | 10.1016/j.jpsychires.2017.01.009; 10.1017/S0016672314000184 |
| TFAM | transcription factor A, mitochondrial | protein coding | 10q21.1 | Chr 10: 58,385,345-58,399,220 | mitochondrial DNA depletion syndrome 15 (hepato-cerebral type) | 20833242 | 10.1016/j.ijdevneu.2010.08.007 |
| BICC1 | BicC family RNA binding protein 1 | protein coding | 10q21.1 | Chr 10: 58,512,872-58,831,435 | Autosomal dominant polycystic kidney disease; renal dysplasia, cystic | 30697050 | 10.2147/NDT.S190048 |
| LOC728640 | family with sequence similarity 133 member C pseudogene | pseudogene | 10q21.1 | Chr 10: 58,715,554-58,716,257 | - | - | - |
| LOC100507008 | long intergenic non-protein coding RNA 844 | ncRNA | 10q21.1 | Chr 10: 58,999,482-59,066,396 | - | - | - |
| RPLP1P10 | ribosomal protein lateral stalk subunit P1 pseudogene 10 | pseudogene | 10q21.1 | Chr 10: 59,088,701-59,089,039 | - | - | - |
| LOC644871 | TNF receptor associated factor 6 pseudogene 1 | pseudogene | 10q21.1 | Chr 10: 59,136,625-59,138,182 | - | - | - |
| PHYHIPL | phytanoyl-CoA 2-hydroxylase interacting protein like | protein coding | 10q21.1 | Chr 10: 59,176,643-59,247,774 | - | - | - |
| FAM13C | family with sequence similarity 13 member C | protein coding | 10q21.1 | Chr 10: 59,246,130-59,363,181 | - | - | - |
| MRPL50P4 | mitochondrial ribosomal protein L50 pseudogene 4 | pseudogene | 10q21.2 | Chr 10: 59,551,404-59,551,878 | - | - | - |
| SLC16A9 | solute carrier family 16-member 9 | protein coding | 10q21.2 | Chr 10: 59,650,764-59,736,002 | - | - | - |
| M1 | Myoregulin | protein coding | 10q21.2 | Chr 10: 59,736,692-59,756,041 | - | - | - |
| CCDC6 | coiled-coil domain containing 6 | protein coding | 10q21.2 | Chr 10: 59,788,747-59,906,556 | Various Cancer | - | - |
| C10orf40 | long intergenic non-protein coding RNA 1553 | ncRNA | 10q21.2 | Chr 10: 59,955,430-59,960,913 | - | - | - |
| ANK3 | ankyrin 3 | protein coding | 10q21.2 | Chr 10: 60,026,298-60,733,490 | ANK3-related intellectual disability-sleep disturbance syndrome; mental retardation, autosomal recessive 37 | 23109352; 27811378 | 10.1002/ajmg.b.32112; 10.18632/oncotarget.13043 |
| ARL4P | ADP ribosylation factor like GTPase 4A pseudogene 1 | pseudogene | 10q21.2 | Chr 10: 60,684,505-60,685,209 | - | - | - |
| CDK1 | cyclin dependent kinase 1 | protein coding | 10q21.2 | Chr 10: 60,778,331-60,794,852 |  | 24564241 | 10.1186/1471-2164-14-S5-S10 |
| RHOBTB1 | Rho related BTB domain containing 1 | protein coding | 10q21.2 | Chr 10: 60,869,438-61,001,440 | - | - | - |
| LOC100507058 | long intergenic non-protein coding RNA 845 | ncRNA | 10q21.2 | Chr 10: 61,016,275-61,026,420 | - | - | - |
| TMEM26 | transmembrane protein 26 | protein coding | 10q21.2 | Chr 10: 61,406,642-61,453,381 | - | 31374203 | 10.1016/j.ajhg.2019.06.012 |
| C10orf107 | ciliary associated calcium binding coiled-coil 1 | protein coding | 10q21.2 | Chr 10: 61,662,929-61,766,766 | - | - | - |
| ARID5B | AT-rich interaction domain 5B | protein coding | 10q21.2 | Chr 10: 61,901,684-62,096,944 | - | 30883267 | 10.2217/pgs-2018-0163 |
| RTKN2 | rhotekin 2 | protein coding | 10q21.2 | Chr 10: 62,183,035-62,268,844 | - | 30883267 | 10.2217/pgs-2018-0163 |
| ZNF365 | zinc finger protein 365 | protein coding | 10q21.2 | Chr 10: 62,374,192-62,480,288 | Narcolepsy type 1 & type 2; Nephrolithiasis | 21853134 | 10.1371/journal.pone.0023450 |
| ALDH7A1P4 | aldehyde dehydrogenase 7 family member A1 pseudogene 4 | pseudogene | 10q21.2 | Chr 10: 62,741,208-62,741,385 | - | - | - |
| ADO | 2-aminoethanethiol dioxygenase | protein coding | 10q21.3 | Chr 10: 62,804,720-62,808,479 | - | - | - |
| EGR2 | early growth response 2 | protein coding | 10q21.3 | Chr 10: 62,811,996-62,919,900 | Charcot-Marie-Tooth disease type 1D & 4E; Dejerine-Sottas syndrome; Neuropathy, congenital hypomyelinating, 1 | 20687139 | 10.1002/ajmg.b.31115 |
| NRBF2 | nuclear receptor binding factor 2 | protein coding | 10q21.3 | Chr 10: 63,133,247-63,155,031 |  | 32245959 | 10.1038/s41537-020-0097-5 |
| JMJD1C | jumonji domain containing 1C | protein coding | 10q21.3 | Chr 10: 63,167,221-63,521,850 | 22q11.2 Deletion Syndrome; Germinoma of the central nervous system | 32610558 | 10.3390/genes11070721 |
| LOC728737 | TatD DNase domain containing 1 pseudogene 1 | pseudogene | 10q21.3 | Chr 10: 63,222,155-63,223,045 | - | - | - |
| MIR1296 | microRNA 1296 | ncRNA | 10q21.3 | Chr 10: 63,372,957-63,373,048 | - | - | - |
| LOC100420046 | PRELID1 pseudogene 3 | pseudogene | 10q21.3 | Chr 10: 63,427,297-63,427,939 | - | - | - |
| LOC84989 | JMJD1C antisense RNA 1 | ncRNA | 10q21.3 | Chr 10: 63,465,229-63,466,563 | - | - | - |
| REEP3 | receptor accessory protein 3 | protein coding | 10q21.3 | Chr 10: 63,521,401-63,625,128 | - | - | - |
| MRPL35P2 | mitochondrial ribosomal protein L35 pseudogene 2 | pseudogene | 10q21.3 | Chr 10: 63,634,317-63,634,827 | - | - | - |
| RPL7AP50 | ribosomal protein L7a pseudogene 50 | pseudogene | 10q21.3 | Chr 10: 63,902,451-63,903,245 | - | - | - |
| LOC100420828 | nuclear pore associated protein 1 pseudogene | pseudogene | 10q21.3 |  | - | - | - |
| LOC645084 | DBF4 zinc finger pseudogene 1 | pseudogene | 10q21.3 | Chr 10: 64,168,959-64,170,850 | - | - | - |
| RPL17P35 | ribosomal protein L17 pseudogene 35 | pseudogene | 10q21.3 | Chr 10: 64,620,374-64,620,922 | - | - | - |
| ANXA2P3 | annexin A2 pseudogene 3 | pseudogene | 10q21.3 | Chr 10: 64,825,572-64,826,579 | - | - | - |
| NEK4P3 | NIMA-related kinase 4 pseudogene 3 | pseudogene | 10q21.3 | Chr 10: 65,054,460-65,055,003 | - | - | - |
| MYL6P3 | MYL6 pseudogene 3 | pseudogene | 10q21.3 | Chr 10: 65,169,438-65,169,869 | - | - | - |
| LOC100421870 | WW domain containing E3 ubiquitin protein ligase 2 pseudogene | pseudogene | 10q21.3 |  | - | - | - |
| LOC100653137 | cadherin-23-like | protein coding | 10q | Chr 10: 67,194,251-67,569,471 | - | - | - |
| CTNNA3 | catenin alpha 3 | protein coding | 10q21.3 | Chr 10: 65,912,457-67,763,637 | Arrhythmogenic right ventricular dysplasia, familial, 13; Familial isolated arrhythmogenic ventricular dysplasia, biventricular form, left dominant form & right dominant form | 23358160; 25664232 | 10.1038/mp.2013.2; 10.1007/s40473-014-0022-1 |
| LRRTM3 | leucine rich repeat transmembrane neuronal 3 | protein coding | 10q21.3 | Chr 10: 66,926,036-67,101,551 | - | - | - |
| LOC100653136 | uncharacterized LOC100653136 | ncRNA | 10q |  | - | - | - |
| RPL7AP51 | ribosomal protein L7a pseudogene 51 | pseudogene | 10q21.3 | Chr 10: 67,334,123-67,334,882 | - | - | - |
