# Supplementary material for: Genome-wide copy number variant screening of Saudi schizophrenia patients reveals larger deletions in cases versus controls
Source: Front Mol Neurosci. 2023 Feb 10;16:1069375. doi: 10.3389/fnmol.2023.1069375 (PMC9950097; doi:10.3389/fnmol.2023.1069375)
Supplement: Supplementary file 2 [file Image_1.pdf]

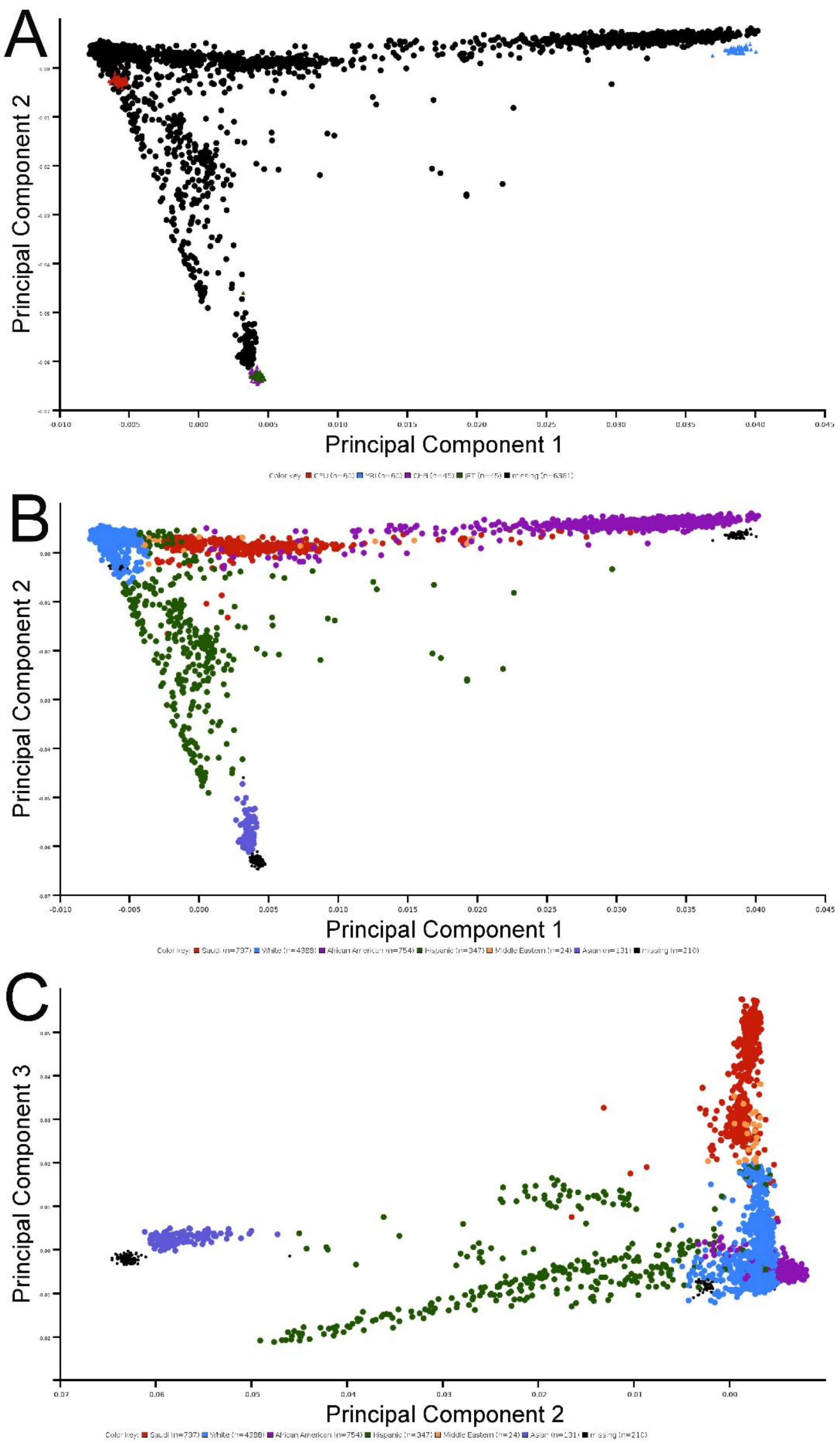

Supplementary Figure 1: Plots of principal components (PC) capturing ancestry showing the relationship between the Saudi samples compared to those of the US controls. Plot A represents PC1 and PC2 anchored by HapMap samples (European ancestry [CEU] in red, African [Yoruban; YRI] in blue, Chinese [CHB] in purple, and Japanese [JPT] in green). Plot B color codes PC1 and PC2 with the imputed ancestry of the US controls overlaid by the Saudi cases and controls in red. Plot C plots demonstrates that PC3 is capturing individuals of Southwestern Asia.
