# Supplementary material for: Genome-wide copy number variant screening of Saudi schizophrenia patients reveals larger deletions in cases versus controls
Source: Front Mol Neurosci. 2023 Feb 10;16:1069375. doi: 10.3389/fnmol.2023.1069375 (PMC9950097; doi:10.3389/fnmol.2023.1069375)
Supplement: Supplementary file 3 [file Image_2.pdf]

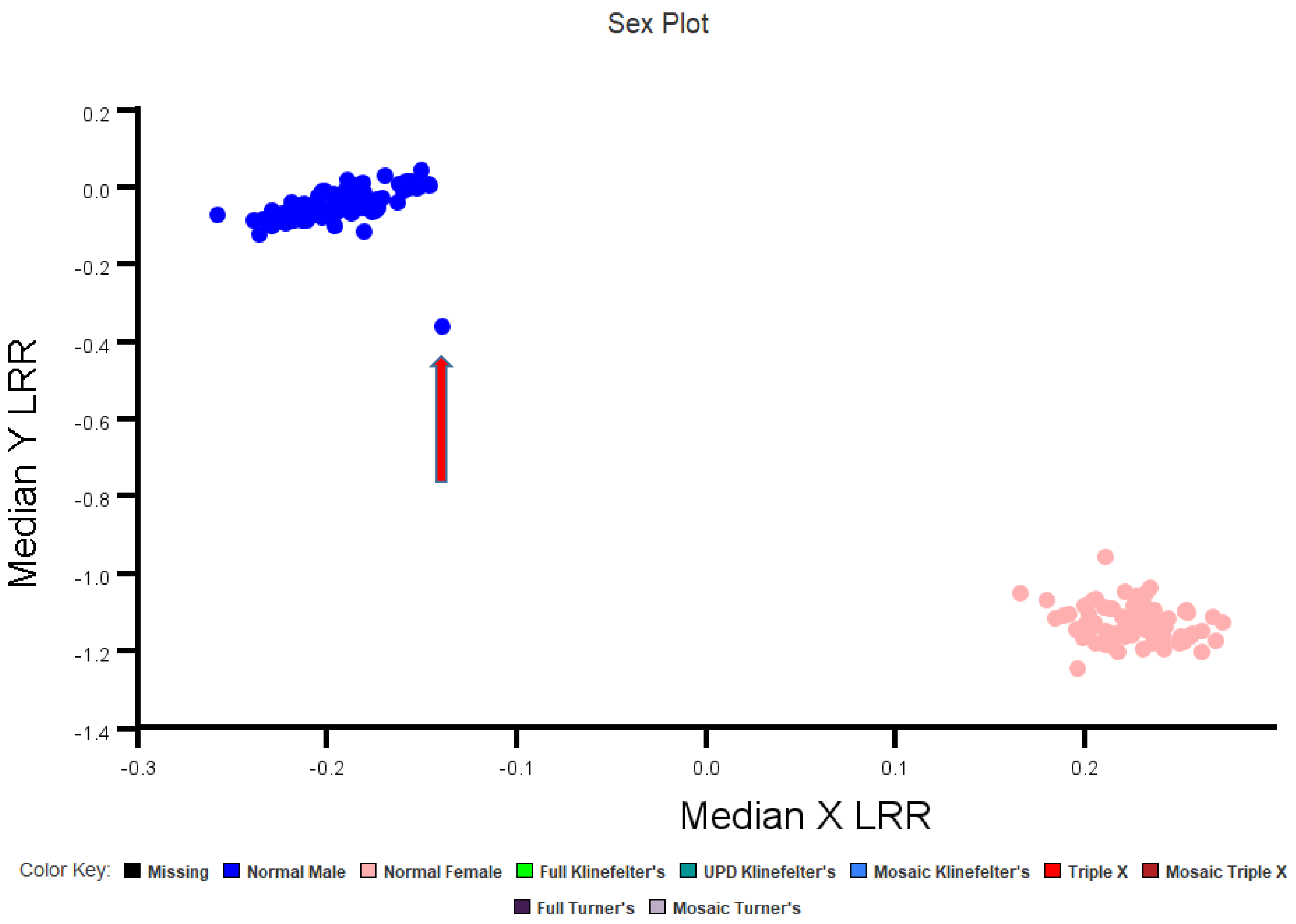

Supplementary Figure 2: Median log R ratio (LRR) values for the X and Y chromosome for all samples. The red arrow indicates a male sample with diminished intensity for Y chromosome.
